# O.3.1-7 Multi-level stakeholders' perspectives on implementation and scaling up community-based physical activity promotion in Germany

**DOI:** 10.1093/eurpub/ckad133.144

**Published:** 2023-09-11

**Authors:** Leonie Birkholz, Philipp Weber, Natalie Helsper, Lea Dippon, Simone Kohler, Klaus Pfeifer, Alfred Rütten, Jana Semrau

**Affiliations:** Friedrich-Alexander-University Erlangen-Nürnberg, Germany; leonie.birkholz@fau.de; Friedrich-Alexander-University Erlangen-Nürnberg, Germany; leonie.birkholz@fau.de; Friedrich-Alexander-University Erlangen-Nürnberg, Germany; leonie.birkholz@fau.de; Friedrich-Alexander-University Erlangen-Nürnberg, Germany; leonie.birkholz@fau.de; Friedrich-Alexander-University Erlangen-Nürnberg, Germany; leonie.birkholz@fau.de; Friedrich-Alexander-University Erlangen-Nürnberg, Germany; leonie.birkholz@fau.de; Friedrich-Alexander-University Erlangen-Nürnberg, Germany; leonie.birkholz@fau.de; Friedrich-Alexander-University Erlangen-Nürnberg, Germany; leonie.birkholz@fau.de

## Abstract

**Purpose:**

Community-based physical activity promotion (c-PAP) with a systems perspective has the potential to address health inequity. To achieve a public health impact, such approaches need to be scaled up. For a successful scale up, a concept is needed that is also scientifically grounded and considers practice-based evidence from various stakeholders at different levels and sectors. Therefore, the aims of this study are to assess what kind of external support communities need for implementation and to identify facilitators and barriers for scaling up c-PAP.

**Methods:**

Two digital workshops were conducted in Germany in March and April 2021. Stakeholders at the community level (n = 163) were involved in the first workshop to identify, what kind of external support communities need and what they can accomplish themselves to implement c-PAP. In the second workshop, stakeholders at the federal and state levels from the sport, health, and social sector as well as scientists (n = 92) discussed facilitators and barriers for scaling up c-PAP interventions. Protocols were compiled and coded using qualitative content analysis.

**Results:**

During the first workshop, we identified 14 themes. They represent external support needs, e.g., guidance and assistance by intermediary organization as well as internal efforts that communities can accomplish themselves, e.g., building local networks. In the second workshop, 11 facilitators and barriers for scaling up c-PAP, e.g., political support or an intermediary organization, were identified.

**Conclusions:**

The identified results provide practice-based evidence on support needed for scaling up, facilitators that promote scaling up and barriers that might hinder scaling up c-PAP in Germany. Various stakeholders emphasized the need for collaboration and partnerships across several sectors as well as changes in current political and legal conditions. In a next step, this practice-based evidence needs to be systematically integrated with scientific-based evidence on key components for scaling up such approaches for the development of an effective scaling up concept.

**Funding Source:**

This research was funded by the Federal Centre of Health Education (BZgA) on behalf of and with funds from the statutory health insurances according to § 20a SGB V in the context of the GKV Alliance for Health.

